# Effect of using knee valgus brace on pain and activity level over different time intervals among patients with medial knee OA: systematic review

**DOI:** 10.1186/s12891-021-04513-0

**Published:** 2021-08-12

**Authors:** Huda Alfatafta, David Onchonga, Mahmoud Alfatafta, lu Zhang, Imre Boncz, Szimonetta Lohner, Bálint Molics

**Affiliations:** 1 Doctoral School of Health Sciences, Faculty of Health Sciences, University of Pécs, Vörösmarty utca 4, 7621 Pécs, Hungary; 2 Biomedical Sciences, University of Strathclyde, Glasgow, UK; 3 Faculty of Health Sciences, Institute for Health Insurance, University of Pecs, Pécs, Hungary; 4 Clinical Center, Medical School, Cochrane Hungary, University of Pécs, Pécs, Hungary; 5 Institute of Physiotherapy and Sport Science, Faculty of Health Sciences, University of Pécs, Pécs, Hungary

**Keywords:** Knee-valgus brace, Pain, Activity level

## Abstract

**Background:**

The Knee valgus brace is one of the accepted conservative interventions for patients with medial compartment knee osteoarthritis to correct the knee varus and increase functional activity level. Nevertheless, comprehensive overview of the effects of using this brace on self-reported pain activity level over time is not available. Thus, this study aimed to systematically review the effect of using this brace on pain and activity levels in the last 20 years in patients with medial compartment knee osteoarthritis.

**Methods:**

Five databases were searched to find articles from the year 2000 to the end of November 2020: Cochrane Central Register of Controlled Trials (CENTRAL), EMBASE, PubMed, Web of Science, and Scopus. Two reviewers independently evaluated the available articles for eligibility and assessed quality. The risk of bias in each study was assessed by two reviewers independently according to the Strengthening the Reporting of Observational Studies in Epidemiology tool (STROBE) for the non-randomized controlled studies and the Cochrane risk-of-bias tool for the randomized controlled studies.

**Results:**

Seven randomized controlled studies and 17 cohort studies (in total 579 participants) were included in the systematic review. Most of these studies found using a knee valgus brace effective in reducing pain and improving activity level over different time intervals. The majority of the included studies (14 studies) evaluated the impact of the brace for a considerably short-term (less than 6 months). Thus, limited evidence is available on the long-term use of the knee valgus brace and its associated complications.

**Conclusion:**

The knee valgus brace is an effective conservative intervention to improve the quality of life and reduce pain during daily activities for some patients. However, the long term of using this brace is still not very convenient, and the patients who benefit most from using the brace should be identified with high methodological quality studies.

**Supplementary Information:**

The online version contains supplementary material available at 10.1186/s12891-021-04513-0.

## Background

Knee osteoarthritis is the most common reason for disability, pain, and limited activity level among the elderly. The medial compartment of the knee joint is 10 times more likely to be affected by osteoarthritis (OA) than the lateral compartment because it receives almost 70% of the total joint load during walking [1–3]. In Europe, it has been seen that 25% of elderly over 50 years have severe knee OA yearly [4], and women are more affected than men (6.6% vs. 4.9%, woman vs. men, respectively) [5]. During medial knee OA, the medial space of the knee joint is narrowing due to cartilage degeneration that leads to a high varus moment [1, 3, 6]. This high varus moment generates pain during daily activity and sometimes during rest in severe cases. Moreover, it has been suggested that patients with knee OA complain from knee instability during daily activities which are correlated with knee pain and low quality of life [7]. Thus, using the knee valgus brace increases the knee's mediolateral stability and reduces the pain [7].

The primary questionnaires that are used to evaluate pain and activity level are the Western Ontario and McMaster Universities Osteoarthritis Index (WOMAC), visual analogue pain score (VAS), the short form 36 (SF-36), and the Knee injury and Osteoarthritis Outcome Score (KOOS). Those questionnaires have high validity and reliability [8–11], and examine the pain and the activity in the last previous week; hence, the patients can remember their experiences with pain and their daily activities [8, 10, 12, 13].

Various interventions (surgical and non-surgical) are recommended based on the Osteoarthritis Research Society International (OARSI) guidelines such as surgical interventions, physiotherapy, orthotics (foot orthoses, knee braces), pain killers, and self-managements. Those interventions aim to reduce pain, improve activity level, and slow disease progressions [14, 15].

The Knee valgus brace is one of the accepted conservative interventions for patients with medial compartment knee OA to improve quality of life and reduce the load on the medial compartment of the knee joint [1, 6]. This brace is used to correct the knee varus through applying valgus force with two methods: bending system (three-point pressure system) directly to the knee joint or through applying valgus force and external rotation of the leg [1, 6, 16, 17]. Both designs aim to reduce the knee varus alignment, unload the medial compartment of the knee and decrease the symptoms [1, 6, 16]. The knee valgus brace could be an off-the-shelf or custom-made brace. Most of the studies recommend using the custom-made knee valgus brace because it shows better fitting, knee varus correction and better activity level improvement [3, 6, 18]. The available systematic review and meta-analysis studies evaluated all kind of knee braces (such as soft, dynamic, valgus, etc.) that are used for patients with medial compartment knee OA, but there is no study evaluate only the effect of knee valgus brace over a different time interval. Thus, the aim of this study is critically evaluating the studies that only assessed the effect of knee valgus brace on pain and activity level among medial knee OA participants in the last 20 years (from 2000–2020). The time interval of using a knee valgus brace will be determined as short-term use (less than 3 months), moderate time use (3–6 months), and long-term use (more than 6 months).

## Methods

The PRISMA (Preferred Reporting Items for Systematic Reviews and Misanalyses) guidelines were used to report the methodology and the results of the systematic review.

### Search strategy

Two independent reviewers searched the following electronic databases from January 2000 until the end of November 2020: Cochrane Central Register of Controlled Trials (CENTRAL), EMBASE, PubMed, Web of Science and Scopus. The used search strategy is available in Additional file 1: Appendix 1 and the search strategy was adapted for the different databases as required.

### Study screening

Two authors independently selected studies based on predefined inclusion criteria. The titles and abstracts were reviewed first, and irrelevant references were excluded. Then full-text publications of potentially relevant studies were obtained and checked for final inclusion. The references and related articles of the selected studies were screened for more suitable studies. Any disagreement was resolved by discussion among the two authors. If they could not reach an agreement, the third author was consulted and a decision was made by a discussion and majority vote. Authors were contacted if the data were not clear or further information were required.

### Eligibility criteria

All studies (randomized-controlled-trials (RCTs), controlled clinical trials (CCTs) and other study designs, such as cohort studies and case–control studies) that evaluated the effects of knee valgus brace on pain and functional activities were included and they have had to be written in English. Also, they had to meet all of the following criteria: (a) adult participants with medial compartment knee osteoarthritis, (b) participants with pain, morning stiffness, and activity level limitations, (c) the outcomes of pain and/or activity level are measured using WOMAC, SF36, KOOS, or VAS, (d) publication between January 2000-end of November 2020.

The study was excluded if (a) it looked at evaluating the knee valgus brace combined with another treatment or medication, (b) studies with children, (c) evaluation the pain and the activity level with other questionnaires, (d) using different kind of knee orthoses instead of knee valgus brace, (e) patients with lateral compartment knee OA or have OA in other joints such as hip or ankle joints. No restrictions if the knee OA is with clinical or/and radiological symptoms.

### Data extraction and risk-of-bias assessment

Two reviewers independently extracted data from the selected studies or reports according to a fixed protocol. The following information was extracted: study design, number of participants, patients’ demographic, the health status of participants, type of knee brace, duration, pain score, activity level scores and funding resources.

The risk of bias in each study was assessed by two reviewers independently according to the Strengthening the Reporting of Observational Studies in Epidemiology tool (STROBE) for the non-randomized controlled studies and the Cochrane risk-of-bias tool for the randomized controlled studies. STROBE evaluates the good reporting of the observational studies and has 22 items to assess the reporting quality of title and abstract, introduction, methods, results and discussion sections [19, 20]. The Cochrane risk-of-bias tool evaluates six items: random sequence generation, blinding of participants and personnel, blinding of outcome assessment, incomplete outcome data, selective reporting and other sources of bias. Each item is judged as being in one of three categories: low (low risk of bias), high (high risk of bias), and unclear (lack of information or uncertainty about the potential for bias). 'Low' indicates a superior quality study, whereas 'high' indicates methodology of inferior quality.

## Results

**Fig. 1:**
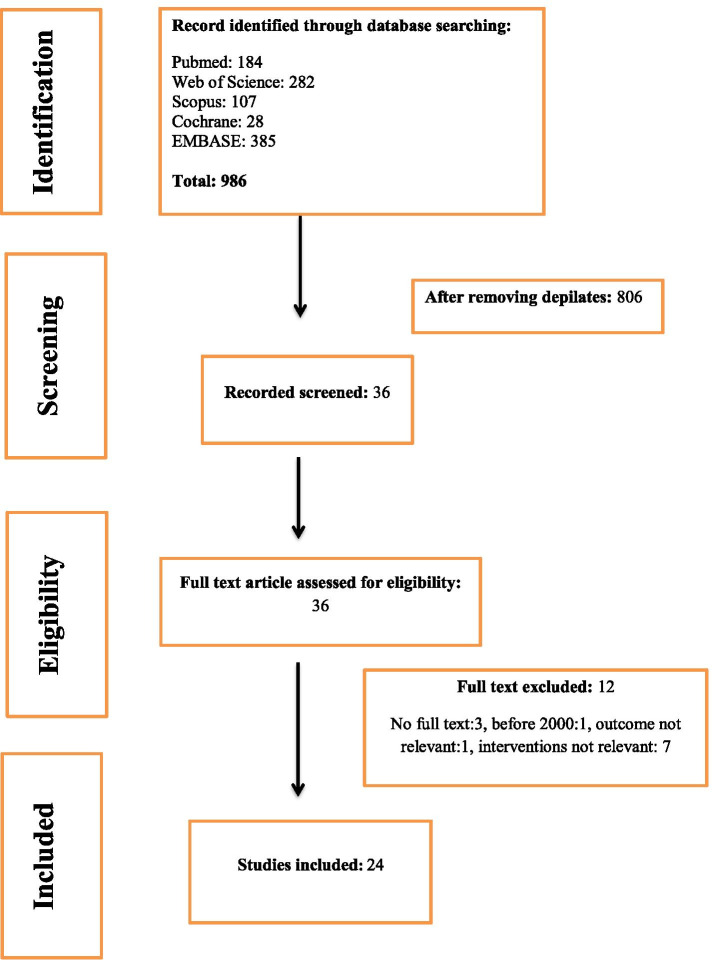
PRISMA flowchart of information through the different phases of a systematic review

A total of 986 potentially relevant records were identified through the systematic literature search of electronic databases. After removing duplicates, 806 unique records were assessed for eligibility (Fig. 1). From these, 770 records were excluded after the title and abstract screening, and another 12 were excluded after full-text screening. Finally, 24 records fulfilled the inclusion criteria ( 579 participants, average age 57 ± 5.5 years, average body mass index 26 ± 2.1 kg/m2). Only seven of them are randomized control studies and the rest either crossover studies or prospective studies (Table 1). Those studies evaluated the effect of knee valgus brace on pain and/or activity level over different time intervals: short term use (less than three months), moderate term use (three to six months), and long-term use (more than six months) among participants with medial compartment knee OA (Table 1).

**Table 1:** Observational and randomized studies on the association of using knee valgus brace and questionnaires (WOMAC, VAS, SF-36, KOOS). ↑ means that a positive, significant change was described in the manuscript between before and after values; – means that the outcomes improved but not significantly; ↓ means that a significant negative change was described in the publications. F: means female, M: means male

First author, publication year (reference no)	Study design	Number of participants	Intervention duration	Type of the knee valgus brace	Used-questionnaire	The direction of effect, as indicated in the manuscript
Jones et al.,2013 [6]	crossover randomized	28 (12f, 16 m)	2 weeks	Donjoy-OA Adjuster, DJO, Vista, USA)	WOMAC VAS pain	↑
Haladik et al. 2014 [1]	prospective cohort	10 (1f, 9 m)	2 weeks	OA Adjuster	WOMAC	↑
Fu et al., 2015 [21]	non-randomized prospective cohort	10 (4f, 6 m)	4 weeks	Unloader valgus knee braces (Ossurhf, Reykjavik, Iceland)	WOMAC VAS pain	↑
Polloet al., 2002 [22]	prospective cohort	11 (1f, 10 m)	2 weeks	Generation II Un-loader ADJ brace, Generation II US	VAS pain	↑
Schmalz et al., 2010 [23]	prospective cohort	16 (8f, 8 m)	4 weeks	Genu Arthro knee brace	VAS pain	↑
Ramsey et al., 2007 [7]	prospective cohort	16 (not available)	2 weeks	GenerationII Unloader Select, Generation II USA, Inc., Bothell, Washington	KOOS	–
Hsieh et al.,2020 [24]	comparative study	20 (13f, 7 m)	1 month and 3 months	Thruster Legacy OA brace	WOMAC VAS pain	↑
Briggs et al. 2012 [25]	prospective cohort	39 (16f,23 m)	3 week, 6 weeks, and 6 months	unloader brace	WOMAC SF36	↑
van Egmondet al., 2017 [26]	randomized controlled trial	100.In Bledsoe Thrustergroup 50 (20f, 30 m).InSofTec group 50 (22f, 28 m)	2 weeks and 12 weeks	the Bledsoe Thrusterbrace (B&Co Inc. N.V., Sint-Antelinks, Belgium) andtheSofTec OA Brace (Bauerfeind AG, Zeulenroda-Triebes, Germany)	VAS pain WOMAC SF-36	↑
Barne et al., 2002 [27]	prospective cohort	30 (12f, 18 m)	8 weeks	Counterforce brace (breg, calif)	SF-36	↑
Thoumie et al., 2018 [28]	randomized controlled trial	32 (24f, 8 m)	6 weeks	The REBELRELIEVER unloading knee brace	VAS pain (100 mm)	↑
Gaasbeek et al.,2007 [16]	prospective cohort	15 (3f, 12 m)	6 weeks	The SofTec OA valgus brace	WOMAC VAS pain	↑
Laroche et al., 2014 [29]	prospective cohort	20 (16f, 5 m)	5 weeks	PROTEOR (France)/ ODRA® brace	WOMAC	↑
Draganich et al.,2006 [18]	Crossover	10 (not available)	5 weeks	Adjustable OA Defiance; dj Orthopedics)	WOMAC	↑
Ornetti et al., 2015 [30]	prospective cohort	20 (16f, 4 m)	6 weeks and 52 weeks	OdrA brace	KOOS VAS pain	↑
Arazpour et al., 2013 [31]	randomized prospective cohort	12 (8f, 4 m)	6 weeks	Custom-made knee valgus brace	VAS pain	↑
RobertLachaine et al., 2020 [17]	randomized crossover	24 (10f, 14 m)	3 months	valgus three-point bending system brace (V3Pbrace), an unloader brace with valgus and external rotation functions (VERbrace) and a stabilizing brace	WOMAC KOOS	↑
Hurley et al., 2012 [32]	prospective cohort	24 (4f, 20 m)	6 months	Breg Fusion valgus unloader braces (custom-made)	WOMAC SF36	–
Iqbal, 2014 [33]	randomized controlled trial	60 (24f, 36 m)	6 months	Custom-made off-loading knee braces	VAS pain (mm) VAS activity (%)	↑
Richards et al., 2005 [34]	crossover study	12 (5f, 7 m)	6 months	GII Orthotics-Europe, Eindhoven, The Netherlands	VAS pain	↑
van Raaij, et al. 2010 [35]	randomized controlled trial	46 (35f, 11 m)	6 months	the MOS Genu1 knee brace	WOMAC Function VAS pain	↑
Ostrander et al., 2016 [36]	randomized controlled trial	16 (8f,8 m)	24 weeks	a medial-unloader brace (Fusion OA; Breg, Inc)	KOOS VAS pain	↑
Hjartarson and Toksvig-Larsen, 2018 [12]	randomized controlled trial	52 out 74 finished one year study	One year	Unloader One® Knee Brace (Ossur,Iceland)	KOOS	–
Sattari&Ashraf,2011 [37]	randomized controlled trial	20 (63%f, 37%m)	9 months	The generation II of knee orthosis	VAS pain	↑

### Short term use (up to three months)

Most of the available studies evaluated the effect of using the knee brace on pain and activity level within short term one month (9 studies), two months (8 studies) and three months (3 studies) (Table 1). All of them support using the knee valgus brace as a conservative intervention for patients with medial knee OA to reduce pain and increase activity level [1, 6, 7, 16–18, 21, 23–26, 28–31].

Within one month, Jones et al. [6] evaluated 28 participants with knee valgus brace and lateral wedge insole. Each condition was used for two weeks with two weeks washout between the two conditions. The results show that a knee valgus brace with a 6-degree knee valgus sitting reduces the pain and improves the activity level significantly (*p* = 0.00) compared to the baseline (no interventions). Fu et al., 2015 [21] also evaluated 10 participants with six different interventions for four-weeks with no wash-out period. The knee valgus brace significantly reduced the pain by 20% in WOMAC and 15.5% in VAS compared to the baseline. Barnes et al., [27] examined 30 patients with medial knee OA for 8 weeks with knee valgus brace and indicated the pain and activity also improved significantly based on the SF-36 questionnaire. Furthermore, 41% of them still use the brace after the investigation, while 35% of them stopped using the brace because of poor fitting or discomfort. After 5 weeks, Briggs et al., Draganich et al. and Laroche et al. [18, 25, 29] studies indicated that the pain and activity significantly improved based on WOMAC and SF-36 in comparison with the no-brace condition. After three months of using the knee valgus brace, both the WOMAC and KOOS scores improved 10–40% on average [17].

In contrast, among these studies, some patients had controversial responses with using the knee valgus brace. In 2007, Ramsey et al. [7] evaluated 16 patients with a neutral brace and 4-degrees knee valgus brace. Each condition was used for two weeks (with two weeks wash-out period between the two conditions). The pain and the activity level were measured using the KOOS questionnaire. The results show that the knee valgus brace could improve pain and activity level but not significantly. This result could be due to the bracing order was not randomized. Moreover, 6 participants (out of 16 participants) complained of a feeling of slipping down the brace [23], and 25% of the participants stop using the brace because they had minor compliance such as redness, blisters, poor fitting, and pain [26]. In further, some users complain form knee flexion limitation during walking with the knee valgus brace which is not very comfortable for them [6, 21, 24, 31].

### Moderate term use (four months to Six Months)

After six months of using the knee valgus brace, positive results were also suggested by six studies (two of them are randomized controlled studies). Briggs et al. [25] study showed that 25% of medial knee OA participants have less pain and only 12 patients had knee surgery after six months of using the knee valgus brace. Moreover, Iqbal, 2014 [33] study assessed Mistry Pakistani patients with medial knee OA for six months with knee valgus brace and found that both the pain and function improved significantly (*p* = 0.00). However knee valgus brace is effective to improve pain and function, five participants of 120 had poor fitting and swelling [33]. Similarly, Richards et al. and Ostrander et al. [34, 36] showed that the knee valgus brace is an effective conservative intervention for carefully selected patients.

In contrast, Hurley et al., 2012 [32] stated that using a knee valgus brace could improve the pain and activity level but not significantly (*p* = 0.05 and *p* = 0.08, respectively) based on WOMAC. This result could be explained by the high body mass index of the participant in that study (31.8 ± 5.2 kg/m2) and a short average brace wearing duration (average 4.7 h per day). In further, van Raaij, et al. [35] also found that patients with knee OA wear the knee valgus brace for few hours per day due to feeling less comfortable.

### Long term use (more than six months)

Only three studies (two of them are randomized controlled trials) evaluated the long-term benefits of using the knee valgus brace between2000-2020 and their results also support using the knee valgus brace (Table 1). Hjartarson and Toksvig-Larsen, 2018[12] evaluated 149 patients with unilateral knee OA who randomly divided into brace group (*n* = 74) and placebo group (*n* = 75). After one year, both groups show improvement in pain and function, but the improvement among the brace group was more significantly based on KOOS (*p* = 0.00). Only 25 participants dropped out from the brace group because they underwent knee surgery or had problems with using the brace. Sattariand Ashraf, [37] ran a randomized controlled study on unilateral knee OA. The participants were randomly divided into three groups: brace group, insole group and control group. After nine months the brace had pain relief compared to the control group (*p* = 0.02). Furthermore, Ornetti et al., 2015 [30] also evaluated their participants after one year of using the knee valgus brace and suggested that 76% of them had significant improvement in pain and activity level (effect size more than 0.8).

### Reporting quality assessment

The Strengthening the Reporting of Observational Studies in Epidemiology tool was used to evaluate the quality of the accepted articles. Concerning the title and abstract, all the accepted studies have informative abstracts that were well reported, except for some studies [7, 16, 23, 27, 29, 34] the abstracts were very brief and not enough information about the results. Regarding the introduction, all of the accepted articles explained the background and the object of the study, except for two studies [18] and Fu et al. [21], the background was brief. In the method section, the study design, participants’ criteria, and data collection process were clearly identified. From the eligible 24 studies, only 10 studies were randomized controlled trials (6 of them with a control group); thus, a high chance of bias was associated with the 14 studies because they did not have a control group or the non-randomized studies. In the result section, the results were well reported in all studies except for two studies [7, 18] they did not mention the details about the recruited participants such as gender or age.

In the discussion, all studies indicated and discussed the key points of the findings. Concerning limitations, all studies stated the limitations, except for [6, 7, 16, 23, 27, 34]. Regarding the source of funding, out of 24 studies,15 studies received external grant and fund and reported the source of the fund and the role of the funders [1, 6, 7, 16–18, 22, 24–30, 36, 38].

For the seven randomized controlled studies, the Cochrane risk-of-bias tool was used (Additional file 1: Appendix 2). The overall biases associated with these results were high especially the performance bias and detection bias as neither the researchers nor the participants were blind about the given interventions.

## Discussion

The available knee orthoses for medial compartment knee OA are numerous. The knee valgus brace is one of the used interventions for patients with medial knee OA to reduce pain and improve activities. This type of brace aims to reduce the knee varus moment through two different mechanisms: applying a three-point pressure system (bending system) directly to the knee joint or through applying valgus force and external rotation. This kind of brace shows better clinical outcomes than soft brace and rest sleeve because of moderate-term reduction of pain and disabilities [38–40],. However, the potential benefits of using this brace are still not clear with low level of evidences. Thus, this study aims to extensively cover the available publications (in the last 20 years) that evaluate the effects of using the knee valgus brace on pain and activity level.

After systematically reviewing the available studies, the outcomes of this study found that the majority of the available studies agree that using a knee valgus brace but with some side effects and fair complications. For instance, Ornetti et al. [30] study found that patients used to wear the brace for more than 8 h per day initially, but then the time of wearing reduced to almost 6 h per day after one year due to pain, discomfort, skin problems, or excessive pressure at the front of the tibia. However, 98.6% of patients have pain relief by using a knee valgus brace [40], some patients stop using the brace due to discomfort, skin irritation, poor fitting, poor appearance, had severe pain that the brace cannot reduce [6, 21, 24, 26, 30, 36].

Moreover, the finding of this investigation noticed that the knee valgus brace could be suitable for some patients more than others. For instance, Barnes et al., [27] suggested that patients who have severe Kellgren-Lawrence grade (KL) grade and higher body mass index 28–30 stopped using the knee valgus brace, whereas patients with lower KL grade (grade II) and BMI between 20–24 still use the brace. Obese participants complain of rotation and skin irritation due to poor fitting. Participants with severe knee OA (KL grade IV) were less satisfied with using the knee valgus brace and found it less effective [6, 24]. Thus, using the knee valgus brace could be more recommended and suitable for the patients who have less than 8 degrees of knee varus, less than 20 degrees knee flexion contracture, mild to moderate knee OA level (KL grade II and III), and their body mass index less than 30 [6, 27, 36].

As a result, it is still important to provide a guideline for orthotists and therapists about the patients' criteria that could fit properly with the knee valgus brace (such as body mass index, pain level, knee varus angel, and other factors). Moreover, it is critical to provide clear information for patients about the duration of wearing and how to deal with related complications. Besides, it is necessary to try the brace on before buying for a few days to avoid disappointment as it is not a cheap intervention.

### The limitations

The used studies for this study have some limitations. Most of the studies had short-term follow-up, a small sample, no control group, and a low level of evidence. Few of them are randomized control studies with a moderate level of evidence. Therefore, it is important to investigate the long-term effect of knee valgus braces with randomized-control studies with high validity questionnaires and high-quality methodology. Additionally, further researches are required to identify the optimal patients who can get the maximum benefit of wearing the knee valgus brace (such as age, gender, BMI, knee varus angle, KL grade, pain level, brace wearing duration).

The limitation of this study was including both the randomized and non-randomized studies. The decision to include all types of studies was due to the limited number of randomized studies that focus on the effect of the knee valgus brace on pain and activity level. Also, it was difficult to include only the randomized studies as they have some dissimilarities in term of control group features, the used questionnaire, the study procedure, and the duration of using the brace. In further, this study focused on evaluating activity level through questionnaires (the self-reported) not by objective methods, such as activity monitors, because mainly using questionnaires is faster, cheaper, and easier for researchers than using activity monitors. However, future studies could be run and include activity level that evaluated my objective methods.

## Conclusion

To sum up, the results of this study found that knee valgus brace could be an effective intervention for specific patients to reduce pain and improve activity level but with fair compliance. However, the long-term effect still not clear, and more researches are required to fill the gaps. This finding could be important for specialists who work with patients with medial compartment knee OA to provide sufficient information about the knee valgus brace for the patients before recommending the knee valgus brace to ensure the best quality of life and pain management.

## Supplementary Information

**Additional file 1:Appendix 1.** Searching Protocol. **Appendix 2.** Risk of bias summary: review authors' judgments about each risk of bias item for each included study.

## Data Availability

Data sharing does not apply to this article as no datasets were generated or analyzed during the current study.
