# Supplementary material for: Effect of using knee valgus brace on pain and activity level over different time intervals among patients with medial knee OA: systematic review
Source: BMC Musculoskelet Disord. 2021 Aug 12;22:687. doi: 10.1186/s12891-021-04513-0 (PMC8362244; doi:10.1186/s12891-021-04513-0)
Supplement: Supplementary file 1 — Additional file 1:Appendix 1. Searching Protocol. Appendix 2. Risk of bias summary: review authors' judgments about each risk of bias item for each included study. [file 12891_2021_4513_MOESM1_ESM.docx]

Appendix 1

Searching Protocol

**#1 osteoarthritis[Title/Abstract] OR osteoarthrosis[ [Title/Abstract]**

**#2 degenerative joint disease[Title/Abstract]**

#3 **osteoarthritis, knee[MeSHTerms]**

#4 #1 OR #2 OR#3

#5 **knee[Title/Abstract]**

**#6 knee joint[MeSHTerms]**

#7 #5 OR #6

#8 **brace*[Title/Abstract] OR bracing[Title/Abstract]**

**#9 orthotic devices[MeSHTerms]**

#10 #8 OR #9

#11 #4 AND #7 AND #10

Appendix 2

Risk of bias summary: review authors' judgments about each risk of bias item for each included study.
